# Absence of Foxp3^+^ Regulatory T Cells during Allergen Provocation Does Not Exacerbate Murine Allergic Airway Inflammation

**DOI:** 10.1371/journal.pone.0047102

**Published:** 2012-10-10

**Authors:** Abdul Mannan Baru, Venkateswaran Ganesh, Jayendra Kumar Krishnaswamy, Christina Hesse, Christopher Untucht, Silke Glage, Georg Behrens, Christian Thomas Mayer, Franz Puttur, Tim Sparwasser

**Affiliations:** 1 Institute of Infection Immunology, TWINCORE, Centre for Experimental and Clinical Infection Research; a joint venture between the Medical School Hannover (MHH) and the Helmholtz Centre for Infection Research (HZI), Hannover, Germany; 2 Department of Clinical Immunology and Rheumatology, Hannover Medical School, Hannover, Germany; 3 Institute for Laboratory Animal Science, Hannover Medical School, Hannover, Germany; Institute of Lung Biology and Disease (iLBD), Helmholtz Zentrum München, Germany

## Abstract

Regulatory T cells (Tregs) play a non-redundant role in maintenance of immune homeostasis. This is achieved by suppressing both, priming of naïve cells and effector cell functions. Although Tregs have been implicated in modulating allergic immune responses, their influence on distinct phases of development of allergies remains unclear. In this study, by using bacterial artificial chromosome (BAC)-transgenic Foxp3-DTR (DEREG) mice we demonstrate that the absence of Foxp3^+^ Tregs during the allergen challenge surprisingly does not exacerbate allergic airway inflammation in BALB/c mice. As genetic disposition due to strain specificity may contribute significantly to development of allergies, we performed similar experiment in C57BL/6 mice, which are less susceptible to allergy in the model of sensitization used in this study. We report that the genetic background does not influence the consequence of this depletion regimen. These results signify the temporal regulation exerted by Foxp3^+^ Tregs in limiting allergic airway inflammation and may influence their application as potential therapeutics.

## Introduction

Regulatory T cells (Tregs), comprising of Foxp3^+^ T cells and Foxp3^−^ Tr1 cells are specialized immune cell populations known to modulate immune responses. The role of Tregs has been well documented in allergies [1]–[5]. They are involved in suppression of allergen-specific T cells, playing an important function in the physiological immune response to allergens. Tregs can also influence B cell responses by modulating IgE production and additionally dampen allergic inflammation by suppressing effector cells like eosinophils, basophils and mast cells [6],[7]. In humans, the ratio of Th2 cells to Tregs has been implicated as a strong determinant of immune outcome to common environmental antigens [1]. As the significant role of Tregs (especially Foxp3^+^ Tregs) becomes evident in modulating allergic responses, new interest in the development of allergy treatments has been brought about by understanding the intricate mechanisms underlying tolerance towards allergens. This involves interventions targeting Tregs both, in allergy prevention and in the therapy of established allergies.

A major bottleneck to specifically understand the role of Foxp3^+^ Tregs has been the lack of specific surface markers that would allow their selective depletion. Hence, former studies characterizing them as CD4^+^CD25^+^ cells have yielded contradictory data [2]–[4],[8]–[11]. This could be attributed to the existence of CD25^−^Foxp3^+^ Tregs which can be found in various organs in mice [12],[13]. Moreover, expression of CD25 on B cells [14], dendritic cells [15] and up-regulation of CD25 on activated conventional T cells [16], can further obscure the results obtained upon depletion strategies employing depleting anti-CD25 antibodies. Furthermore, this poses a potential limitation in assessing the influence of Tregs during the various phases of allergic inflammation, as the depleting anti-CD25 antibodies persist *in vivo* for more than two weeks [17] and might thus interfere with both, the developing and the established Th2 responses. Concomitantly, forkhead box transcription factor 3 (Foxp3) has been identified as a master regulator of Treg function and a specific marker for murine CD4^+^ Tregs [18]. Broader expression of Foxp3 on other cells, like epithelial cells [19] and macrophages [20] has been reported. However, these observations could not be reproduced independently, confirming a T cell intrinsic function of Foxp3 [21]–[24].

To address the role of Foxp3^+^ Tregs in allergic airway inflammation, we made use of BAC-transgenic DEREG (DEpletion of REGulatory T cells) mice. DEREG mice express the high affinity diphtheria toxin receptor (DTR)-eGFP fusion protein under the control of the Foxp3 promoter, allowing both viable isolation and inducible depletion of Foxp3^+^ Tregs [13]. As cessation of DT administration to DEREG mice results in replenishment of Tregs to almost normal levels in about a week’s period, they present a suitable tool to address the role of Tregs at various phases of allergic airway inflammation [25]. In this study we aimed to specifically address the involvement of Foxp3^+^ Tregs in curtailment of allergic inflammation during an active allergen provocation. This reflects a more clinically relevant setting, considering that sensitization to the allergen to have already occurred, and thus represent a Foxp3^+^ Treg mediated potential therapeutic approach. Surprisingly, absence of Foxp3^+^ Tregs during the allergen challenge did not result in further aggravation of the inflammatory response and pathology of the lungs. We further confirmed this observation to be independent of the genetic background. These results highlight the temporal regulation exerted by Foxp3^+^ Tregs and their stronger influence on sensitization phase of allergic airway inflammation.

**Figure 1:**
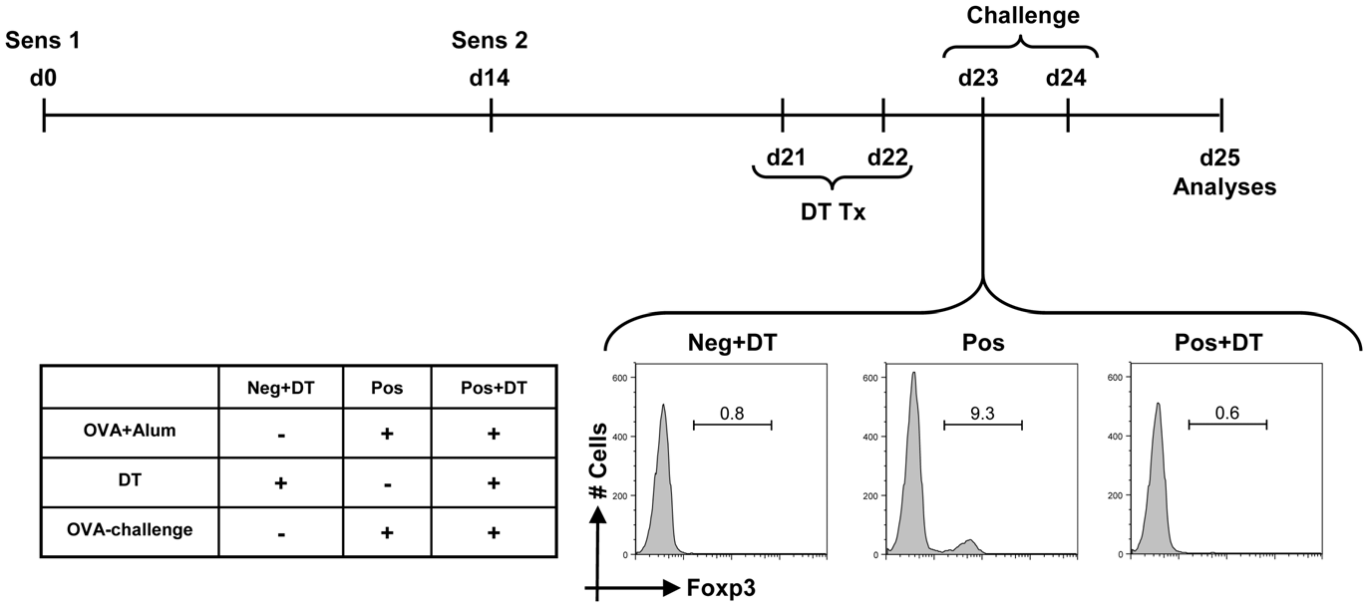
Schematic representation of the experimental set-up. DEREG-BALB/c mice were sensitized to OVA in presence of alum on day 0 and day 14. Foxp3^+^ Treg depletion was achieved by intra-peritoneal administration of diphtheria toxin (DT) a week post the last sensitization for two consecutive days. Mice were bled through retrobulbar venous plexus one day after the last DT treatment and were analyzed for efficient depletion of Foxp3^+^ Tregs. Negative control (Neg+DT) mice were sham sensitized with PBS in alum and were also treated with DT to rule out any unspecific inflammatory effects. Positive control (Pos) mice were challenged with intra-nasal application of OVA for two consecutive days without any DT treatment. The experimental group of mice (Pos+DT) were challenged with OVA for two consecutive days 24 hr post last DT treatment. Histograms demonstrate the frequency of Foxp3^+^ cells on gated live CD4^+^ T cells, from one representative mouse of each group.

## Methods

### Mice

DEREG mice were bred at the animal facility of Twincore (Hannover, Germany) and at the Helmholtz Centre for Infection Research (HZI, Braunschweig, Germany). 6–12 weeks old sex and age matched mice were used. All animals were housed under specific pathogen-free conditions. Mice were sacrificed by intra-peritoneal injection of ketamine-hydrochloride and xylazin-hydrochloride as approved by German animal welfare law. All mouse experiments conducted were in accordance to the described procedures in ethics applications approved by the institutional animal welfare and by the local government namely the Lower Saxony State Office for Consumer Protection and Food Safety (approval reference number: 33.9-42502-04-11/0453). Every effort was made to minimize any sort of suffering to the animals.

**Figure 2:**
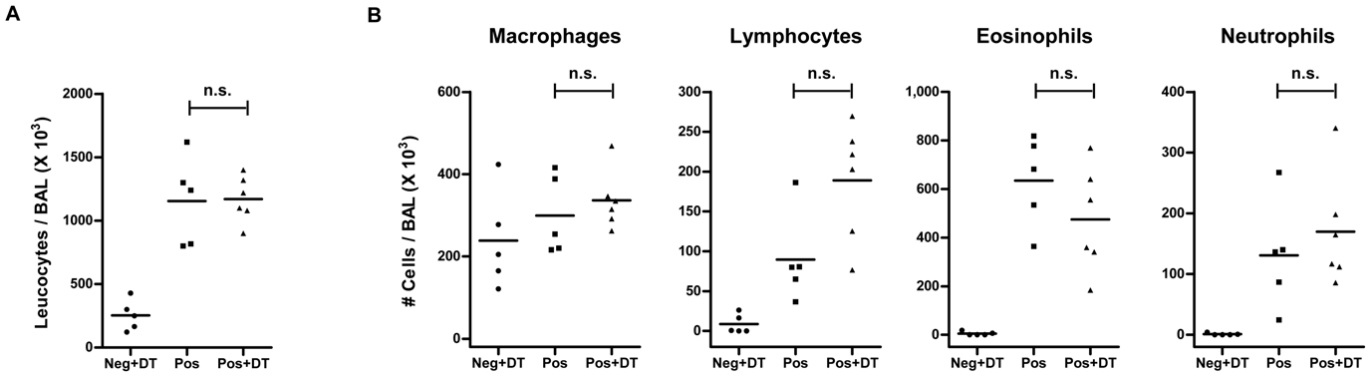
Total cellular infiltration and eosinophilia in BAL remain unaffected by absence of Foxp3^+^ Treg during allergen challenge. (A) Total cellular infiltration in the BAL was determined by counting live cells using trypan blue exclusion dye under light microscope. Each dot represents data from individual mice. Horizontal bar indicate the mean. Data shown is a representative of three individual experiments using 4–10 mice in each group. (B) Differential cellular count was done on Diff-Quik stained slides of infiltrating BAL cells by standard morphological parameters in a single blinded manner. A total of 200 leucocytes were counted from random fields. Total numbers of eosinophils, macrophages, lymphocytes and neutrophils are shown. Each dot represent individual mouse. Horizontal bar indicates the mean. Data shown is a representative of three individual experiments using 4–10 mice in each group. Mann Whitney test was used to determine statistical significance. n.s. = non-significant.

### Ovalbumin-induced Allergic Airway Inflammation

DEREG-BALB/c mice were sensitized with 10 µg OVA (grade VI) (Sigma-Aldrich, Munich, Germany) adsorbed on 2 mg Imject® Alum (Thermo Scientific, Illinois, USA) via i.p. administration on day 0 and day 14. Besides these two sensitizations, an additional booster injection was given in case of DEREG-C57BL/6 mice on day 21 to attain optimal sensitization. Treg depletion was achieved by i.p. administration of 0.5 µg or 1 µg DT (Calbiochem, Darmstadt, Germany) one week post last sensitization to DEREG-BALB/c or DEREG-C57BL/6 mice respectively. Positive control (Pos) and experimental (Pos+DT) group of mice were challenged intra-nasally under isofluran-induced anesthesia with 30 µg of OVA (grade V) (Sigma-Aldrich, Munich, Germany) in PBS on two consecutive days. Control mice were sham-sensitized with PBS+Alum and DT treated (Neg+DT).

**Figure 3:**
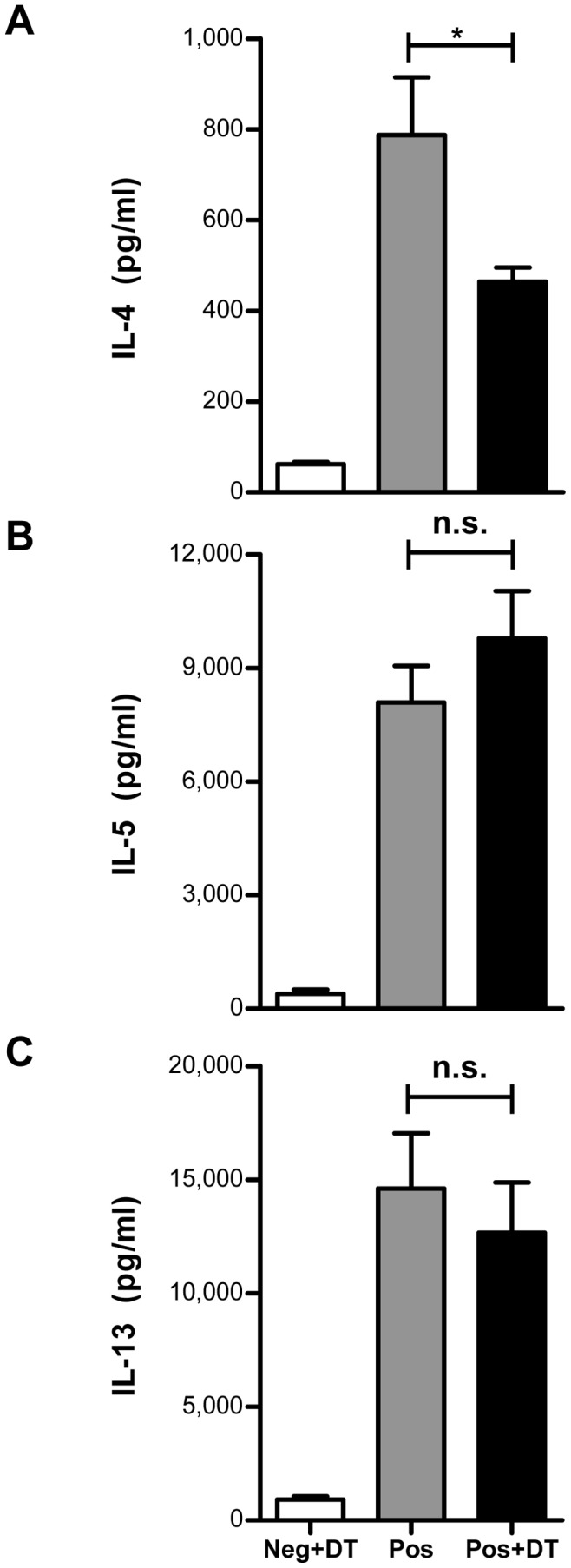
Th2 cytokines secretion after selective elimination of Foxp3^+^ Tregs. Lung draining mediastinal lymph node cells were pooled from each group of mice (n = 4–10) and stimulated *ex vivo* with OVA. 72 hr post stimulation cell free supernatants were collected and analyzed for Th2 cytokines (IL-4, IL-5 and IL-13) by ELISA using matched antibody pairs. Data shown are a representative of three individual experiments. Histograms represent mean values and error bars represent SD of ELISA replicates performed on four individual stimulations from each group. Mann Whitney test was used to determine statistical significance. *p≤0.05 and n.s. = non-significant.

### Bronchoalveolar Lavage (BAL) and Differential Cell Count

Animals were sacrificed 24 hours post last challenge by intra-peritoneal injection of 6.25 mg ketamine-hydrochloride and 0.75 mg xylazin-hydrochloride per mouse, and their trachea was cannulated. Airways were flushed with 0.8 mL ice cold PBS (3X). Total BAL cells were counted using trypan blue exclusion dye after lysing RBCs. 5–10×10^4^ cells in 100 µL PBS were used for cytospots (700 rpm, 5 min; Cytospin3; Shandon). Slides were stained with Diff-Quik kit (Medion Diagnostics, Germany) according to the manufacturer’s protocol and a total of 200 leucocytes were differentially counted under light microscope from random fields.

**Figure 4:**
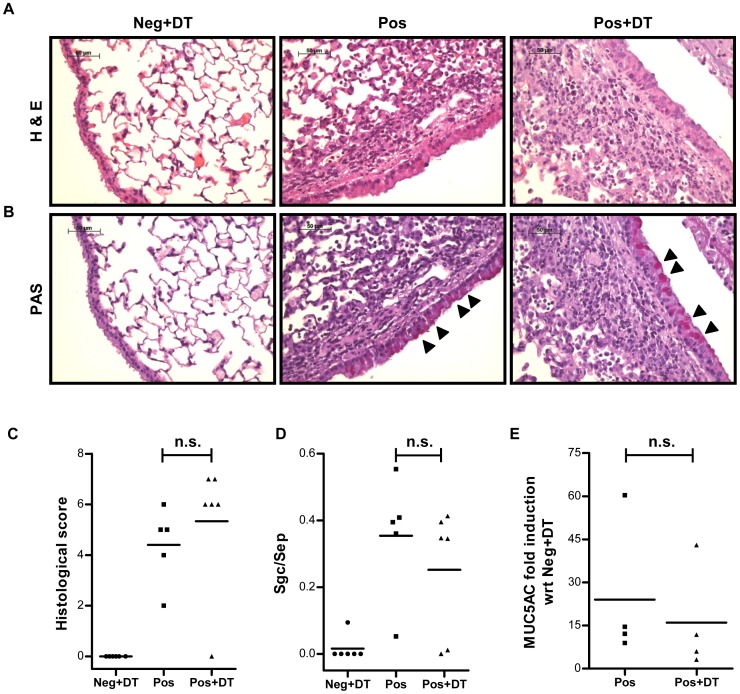
Qualitative and quantitative histological analysis of the lungs. Formalin fixed lung tissues were paraffin embedded and sections of 3 µm were cut. These sections were stained with H&E (A) or PAS (B) dyes to determine cellular infiltration and mucus production in lung, respectively. (B) Arrow heads indicate mucus producing goblet cells. (C) Overall lung extension, peri-bronchial and peri-vascular cellular infiltration and interstitial edema were considered as parameters for histological scoring in a double blinded manner. (D) Mucus secretion was assessed by measuring the surface area of mucus-containing goblet cells (Sgc) per total surface of airway epithelial area (Sep) measured. (E) The expression of the MUC5AC gene was quantified using specific primers in a quantitative PCR. Fold increase in the expression of MUC5AC in the positive control (Pos) and experimental (Pos+DT) group are plotted with respect to expression levels observed in the negative control (Neg+DT) group. Expression of GAPDH mRNA was used as internal control for data normalization. Data shown is a representative of three individual experiments using 4–10 mice in each group. Mann Whitney test was used to determine statistical significance. n.s.  =  non-significant.

### Analysis of the Cytokine Secretion

Mediastinal lymph nodes were pooled from all the mice from each group and single-cell suspensions were obtained by mechanical disruption. A total of 1×10^6^ cells were seeded in 96-well U-bottom plates in 200 µL complete RPMI medium (Gibco, Darmstadt, Germany) containing 100 µg/mL OVA (Grade VI). Culture supernatants were harvested after 72 hr and frozen at −80°C until further use. IL-4, IL-5 and IL-13 cytokines were measured in cell-free supernatants by ELISA using matched antibody pairs purchased from R&D systems (R&D, Wiesbaden-Nordenstadt, Germany). ELISA assays were performed according to the manufacturer’s protocol.

**Figure 5:**
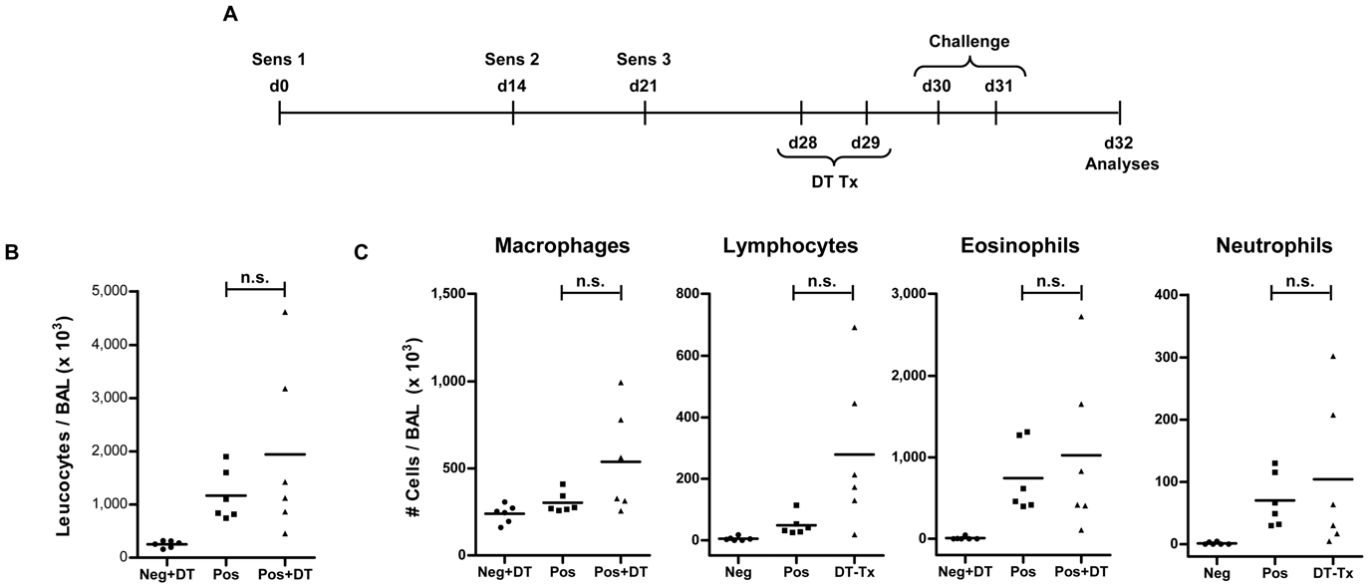
No influence of genetic susceptibility on the effect of Treg depletion during allergen challenge. (A) DEREG-C57BL/6 mice were sensitized to OVA with Alum on day 0, day 14 and day 21. One week post last sensitization DT was administered to mice i.p. Mice were challenged with 30 µg OVA via intra-nasal route for two consecutive days and analyzed 24 hr post last challenge. (B) Total cellular infiltration in BAL was determined by live cell counting using trypan blue exclusion dye. (C) Differential cell count was performed on Diff-Quik stained cytospots made from BAL cells. 200 cells were counted from random fields under light microscope in a single blinded manner. Data shown is a representative of three individual experiments using 3–6 mice in each group. Each dot represents data from individual mouse. Horizontal bar indicate mean value. Mann Whitney test was used to determine statistical significance. n.s.  =  non-significant.

### Quantitative PCR

The left lobe of the lung was frozen immersed in TRIzol (Invitrogen, Darmstadt, Germany) at −80°C till further use. Total RNA was isolated according to the manufacturer’s protocol and quantified using the NanoDrop-1000 spectrophotometer (Peqlab, Erlangen, Germany). cDNA was synthesised using Oligo d(T) primers and Fermentas Revert Enzyme kit (Fermentas, St. Leon-Rot, Germany) using 1 µg total RNA as template. Quantification of MUC5AC gene was done by qPCR using the primers (5′cttcaacggcagtccaaaat 3′) and (5′ctcaaggggtgtcagcctaa 3′) (Eurofins MWG Operon, Ebersberg, Germany). GAPDH was used as an internal control. SYBR green mix and primers for GAPDH were obtained from SAbiosciences (Hilden, Germany). qPCR reaction and data analysis was carried out using Lightcycler 480 (Roche, Penzberg, Germany).

### Lung Histology and Quantification

The lungs were fixed by intra-tracheal instillation of 4% buffered paraformaldehyde, ligated and stored immersed in fixative. Specimens were trimmed according to the industrial guidelines for standardized tissue trimming RITA [26]. Uniform samples were embedded into paraffin, and 3 µm sections were stained with hematoxylin and eosin (H&E) or periodic acid-schiff (PAS) stain to estimate cellular infiltration and airway epithelial mucus production, respectively. The surface area of mucus-containing goblet cells (S_gc_) per total surface area of airway epithelial basal membrane (S_ep_) were determined using a computer-assisted tool (Axio Vision 4.8 Carl Zeiss).

### Statistical Analysis

Data are represented as means + standard deviation (SD). Comparative groups were tested for statistical significance by Mann-Whitney U test using Prism 5 software. *p* values less than 0.05 were considered statistically significant. n.s.  =  non-significant, *p≤0.05 and **p≤0.01.

## Results

### Absence of Foxp3^+^ Tregs during Allergen Challenge does not Augment Cellular Infiltration in the Lungs

To study the temporal influence of Tregs we employed standard OVA/alum model of murine experimental allergic airway inflammation in DEREG mice and adopted distinct regimens for BALB/c or C57BL/6 strains of mice. These regimens were standardized in both the strains to respectively mimic induction of an apparent airway inflammation but concurrently not to reach a threshold response, in order to monitor the influence of Treg depletion. Also the dosage of DT in both the strains of mice was titrated to achieve maximum Treg depletion avoiding any non-specific side-effects, especially in BALB/c mice which are relatively more sensitive to DT than C57BL/6 mice.

To analyze the influence of absence of Tregs during the allergen challenge, we administered DT to DEREG-BALB/c mice after the completion of sensitization and just prior to the initiation of intranasal allergen challenges (Fig. 1). Twenty four hours after the last DT treatment, mice were bled through retrobulbar venous plexus to confirm ablation of Foxp3^+^ Tregs. More than 90% reduction in the frequency of CD4^+^Foxp3^+^ Tregs was observed in all DT treated mice (Fig. 1). Although Foxp3^+^ Treg depletion was demonstrated in blood, a comparable depletion in the target organ i.e. lung needed to be confirmed. As the sensitized and DT treated mice were to be used for continuation of the experiment, we used another set of mice to address this issue. DT was administered to DEREG mice on two consecutive days and the mice were sacrificed on the following day. The blood and lungs were analyzed in parallel to address if decrease in the frequency of Foxp3^+^ Tregs from blood reflects the depletion in lungs too. We observed ablation of Foxp3^+^ Tregs to similar frequencies in both blood and lungs confirming that the regimen used here successfully depleted Foxp3^+^ Tregs also in the target organ (Fig. S1).

Subsequently, intra-nasal OVA challenges were performed in sensitized mice and they were sacrificed 24 hrs post last challenge. To validate the effect of absence of Foxp3^+^ Treg only during the allergen challenge, we collected sera from individual mice and confirmed comparable sensitization in both the groups of sensitized mice by performing antigen-specific ELISA for IgE and IgG1 antibodies (Fig. S2).

All mice that were sensitized and challenged with OVA (Pos and Pos+DT) showed enhanced cellular infiltration in the BAL when compared to the sham-sensitized group of mice (Neg+DT) (Fig. 2A). The BAL of OVA challenged mice indicated enhanced frequencies and total numbers of lymphocytes and granulocytes (mainly eosinophils) in comparison to the un-challenged mice (Fig. 2B). Surprisingly, OVA-sensitized DEREG-BALB/c mice depleted of Tregs (Pos+DT) during the allergen challenge showed no significant alteration in total cellular infiltration in BAL as compared to sensitized mice with an intact Treg compartment (Pos) (Fig. 2A). Differential cell count revealed no significant change in frequencies of various populations infiltrating the broncho-alveolar spaces, namely eosinophils, macrophages, lymphocytes or neutrophils (Fig. 2B).

A direct inflammatory influence of DT was already excluded by administering DT to negative control mice (Neg+DT). Since negative control mice were only sham-sensitized, additive influence of DT in OVA-sensitized mice was not ruled out. To analyze this likelihood we used OVA-sensitized wild type mice and administered DT before allergen challenges. All the parameters analyzed for inflammation and lung pathology in wild type mice were similar to those observed in DEREG mice without DT treatment (data not shown). Thus, specific absence of Foxp3^+^ Tregs during allergen challenge did not influence cellular infiltration in the lungs.

### Absence of Tregs during the Allergen Challenge only Moderately Affects the Allergen-specific Th2 Cytokines

To investigate if the absence of Tregs during allergen challenge had any influence on Th2 cytokine production, we stimulated the lung draining mediastinal lymph node cells with OVA and analyzed the cell free supernatants for secreted Th2 cytokines by ELISA. Quantification of IL-5 (Fig. 3B) and IL-13 (Fig. 3C) demonstrated comparable levels of cytokine in both the groups of DEREG mice which did or did not receive DT treatment before allergen challenges (Pos and Pos+DT). Surprisingly, mice challenged in absence of Foxp3^+^ Tregs demonstrated slightly reduced levels of IL-4 (Fig. 3A). However, the functional relevance of diminished IL-4 production could not be translated into an observable reduction in inflammatory response.

### Treg Depletion before Allergen Challenges does not Exacerbate Lung Pathology

As induction of airway inflammation results in characteristic modification of lung tissue in terms of cellular pathology and goblet cell hyperplasia, we monitored these features by histological examination and quantitative PCR (qPCR). The lung sections were stained with hematoxylin-eosin (H&E) (Fig. 4A) or Periodic Acid Schiff (PAS) stain (Fig. 4B) for determination of cellular infiltration plus lung pathology and goblet cells hyperplasia, respectively. Double blinded quantitative histological analysis performed on the basis of overall lung extension, peri-bronchial and peri-vascular cellular infiltration as well as interstitial edema on H&E stained slides revealed no statistically significant differences in the overall score from lungs of Treg depleted (Pos) or non-depleted (Pos+DT) mice (Fig. 4C). Similarly, no significant differences were observed upon quantification of mucus secretion as assessed by determining the ratio of surface airway epithelial area of mucus containing goblet cells (Sgc) to total airway epithelial area measured (Sep) on PAS stained slides from lungs of DT treated or untreated mice (Fig. 4D). Additionally, mucus production was quantified by real time PCR of MUC5AC gene on cDNA prepared from lung tissues. In line with the histology results, the expression level of MUC5AC gene in lungs from Treg depleted (Pos+DT) or non-depleted (Pos) DEREG mice groups were comparable (Fig. 4E). Thus, absence of Foxp3^+^ Tregs during allergen provocation did not result in aggravated lung pathology.

### Genetic Susceptibility does not Influence Airway Inflammation upon Depletion of Foxp3^+^ Tregs during Allergen Provocation

Genetic differences are known to strongly influence the predisposition towards allergies [27]–[29]. The study by Lewkowich *et al.* demonstrated that ablation of Tregs using depleting anti-CD25 antibodies lead to a severely aggravated airway inflammation in a comparatively allergy resistant (C3H) strain of mice, whereas only a slight enhancement in inflammation was observed in an allergy sensitive strain of mice (A/J) upon similar treatment [10]. We thus next explored, whether a stronger importance of Foxp3^+^ Treg depletion during the allergen provocation phase could be observed in C57BL/6 mice, which are relatively less susceptible to allergy. Interestingly, DEREG mice on C57BL/6 background also demonstrated no exacerbation in lung inflammation upon allergen challenge in the absence of Tregs (Fig. 5). Contrarily, depletion of Tregs during the sensitization phase resulted in severe exacerbation of lung pathology in mice on both, BALB/c (Fig. S3) and C57BL6 genetic background [5].

## Discussion

Comprehending the temporal influence of Tregs in development and progression of allergic responses can have strong implications for their therapeutic exploitation. In this study we analyzed the influence of Foxp3^+^ Tregs during the allergen provocation in a murine model of experimental allergic airway inflammation. Surprisingly, the absence of Tregs during the allergen challenge in sensitized DEREG-BALB/c mice did not result in a significant augmentation of inflammation or lung pathology.

Strain specific differences have been observed in murine models of experimental allergic airway inflammation [30]–[31]. In the study by Lewkowich *et al.*, depletion of Tregs upon administration of depleting anti-CD25 antibodies resulted in enhanced cellular infiltration and eosinophilia in BAL of both, an allergy-resistant and an allergy-susceptible strain of mice [10]. In congruence, we also observed elevated cellular infiltration and eosinophilia in BAL of DEREG mice upon Treg depletion during ongoing sensitization on both, BALB/c (Fig. S3) and C57BL/6 [5] genetic backgrounds. Nevertheless, use of depleting anti-CD25 antibodies did not permit to discriminate the consequences of Treg ablation during the sensitization versus the effector phase. Here, we demonstrate that although both BALB/c and C57BL/6 strains of mice show elevated inflammatory responses upon depletion of Tregs during the sensitization phase, neither strain showed an aggravated response in the absence of Foxp3^+^ Tregs during the allergen challenge, highlighting the significant temporal influence of Foxp3^+^ Tregs.

Many studies have correlated induction and expansion of Tregs to amelioration of allergic airway inflammation. Oral administration of bacterial products has been shown to prevent asthma via recruitment of Foxp3^+^ Tregs [32]. By using a gastrointestinal nematode *Heligmosomoides polygyrus,* Wilson *et al*. reported a Treg-dependent protection in mice which was reversed upon their depletion during the effector phase [33]. Contrarily, in a murine model of experimental allergic airway inflammation, protection provided by the filarial parasite *Litomosoides sigmodontis* was not reversed by depletion of Tregs upon administration of anti-CD25 antibodies *in vivo*, although an increase on Tregs was evident upon infection [34]. Also, infection with *Acinetobacter baumannii* ameliorates allergic airway inflammation in a Foxp3^+^ Treg-independent manner [35]. Furthermore, Foxp3^−^ Tr1 cells that produce immunosuppressive IL-10 have been strongly implicated in modulating allergic responses [1],[36]. Concomitantly, IL-10 plays a non-redundant role in maintaining tolerance at mucosal surfaces including the lungs [37]. Altogether these studies implicate multiple regulatory mechanisms to maintain functional tolerance to allergens, working in parallel with Foxp3^+^ Tregs.

It is thus tempting to speculate that complete absence of, or attenuated Foxp3^+^ Treg responses during early phases of antigen priming (sensitization) could result in a prominent disposition to develop allergies. However, temporary absence of Tregs later during the antigen provocation plays a relatively inconspicuous role. In line with this, the colonization by commensals in early life has been recently suggested to restrict iNKT cell activation and thereby to prevent mucosal pathologies including allergic lung inflammation [38]. However, in this study the involvement of Foxp3^+^ Tregs as a potential mechanism of action remains to be determined. Interestingly, the colonization of adult germ-free mice could not rescue the phenotype, illustrating that the time window of this regulation might be very critical.

Although the absence of Foxp3^+^ Tregs during the allergen provocation does not further exacerbate the lung pathology, this does not potentially exclude that allergic inflammation can be tamed by therapeutic augmentation of Foxp3^+^ Treg numbers or by administration of functional Tregs during the allergen challenge phase. Indeed studies have established protection from lung inflammation and pathology upon transfer of Tregs during the effector phase of allergic airway inflammation [39]–[41]. Consequently, Tregs remain to be promising candidates for therapeutic immune interventions in established allergies. In addition, we propose that the role of Foxp3^+^ Tregs at inhibiting allergic responses under physiological conditions is clearly less pronounced during the allergen provocation phase as compared to during the sensitization phase.

## Supporting Information

Figure S1 **Depletion of Foxp3^+^ Tregs in lungs of DT treated mice.** Since the sensitized and DT treated mice were to be challenged and as the lungs from these mice were used for mRNA extraction and histology, we used another set of mice to confirm depletion of Foxp3^+^ Tregs in lungs. DEREG-BALB/c mice received two i.p. injections of 0.5 µg DT on consecutive days and were sacrificed post 24 hrs of last treatment. Blood and lungs were collected and analyzed by FACS for Foxp3^+^ Tregs. Lungs were digested with collagenase D (Sigma-Aldrich, Munich, Germany) and DNAse I (Roche, Penzberg, Germany) and after RBC lysis blood and lung cells were stained for CD3, CD4 and intra-cellular Foxp3. Equivalent depletion of Foxp3^+^ Tregs was observed in lungs and blood as shown in the representative FACS histograms gated on live CD3^+^CD4^+^ T cells. Each dot in the graph represents data from individual mouse and horizontal bar indicate mean value. Data shown is a representative of two individual experiments with 3 mice per group.
(TIF)

Figure S2 **Sensitization remains unaffected by Foxp3^+^ Treg depletion during allergen provocation.** To comprehend the influence of the absence of Tregs during the allergen challenge, it was essential to confirm an equivalent sensitization of mice in both the sensitized groups (Pos and Pos+DT). Individual sera samples were used to determine the titer of OVA-specific antibodies by ELISA. To measure OVA-specific IgE and IgG1, 10 µg/mL OVA (Grade V) (Sigma-Aldrich, Munich, Germany) was coated over night onto Maxisorp microtiter plates (Nunc, Roskilde, Denmark). After blocking with 1% BSA in PBS, samples and standards {IgG1: clone OVA-14 (Sigma-Aldrich, Munich, Germany) and IgE: clone-2C6 (AbD-Serotec, Oxford, UK)} were added and incubated over-night. Detection limits were 1.56 ng/mL and 3.125 ng/mL for IgE and IgG1, respectively. Bound antibodies were probed with biotinylated anti-mouse IgG1 (A85-1, BD Pharmingen, Heidelberg, Germany) or anti-mouse IgE (R35-92, BD Pharmingen, Heidelberg, Germany) for one hour at 37°C. Streptavidin conjugated alkaline phosphtase (Strep-AKP; BD Pharmingen, Heidelberg, Germany) was used to detect biotinylated antibodies. ELISA was developed with phosphatase substrate (Sigma-Aldrich, Munich, Germany) at room temperature. Absorbance was measured on the BioTek ELISA reader (Bad Friedrichshall Germany) at 450 nm with 570 nm filter as reference. Significant titers of OVA-specific IgE and IgG1 antibodies were detected in both the groups of mice sensitized to OVA in the presence of alum (Pos and Pos+DT). Antigen-specific IgE and IgG1 titers were at comparable levels irrespective of the Treg depletion status. As the sera were collected post allergen challenges, equivalent antigen-specific immunoglobulin levels validates that depletion of Tregs during allergen provocation did not affect the sensitization in the time frame of measurement i.e. from DT treatment to analysis. This further substantiates that the data presented in this study reflects the influence of the absence of Tregs only during the allergen challenge. Data are depicted as mean + SD and are representative of five individual experiments with 4–10 mice per group. Mann Whitney test was used to determine statistical significance. n.s. = non-significant.
(TIF)

Figure S3 **Depletion of Tregs during the sensitization phase exacerbates pathology in DEREG-BALB/c mice.** We have recently reported that depletion of Tregs during the sensitization phase of allergic airway inflammation exacerbates lung pathology significantly using DEREG mice on C57BL/6 background. To assess the influence of strain specificity in absence of Tregs during the priming phase, we performed Treg ablation in DEREG mice on BALB/c genetic background during the sensitization phase. As BALB/c mice are comparatively allergy sensitive they were sensitized to OVA with alum by two i.p. injections one on day 0 and another on day 14. 0.5 µg DT was administered intra-peritoneally on two consecutive days after each sensitization to achieve Foxp3^+^ Tregs depletion during the sensitization phase. Mice were rested for one week after DT treatment, and then bled through retrobulbar venous plexus to confirm the rebound of Foxp3^+^ Tregs. Consequently, mice were challenged with OVA via intra-nasal route for two consecutive days and analyzed one day post last challenge. BAL was collected through the cannulated trachea with 0.8 ml PBS (3x). (A) Total cellular infiltration was estimated in the BAL by live cell counting with trypan blue exclusion dye. Each dot represents data from individual mice and horizontal line depicts mean value. (B) 5–10×10^4^ BAL cells were spotted on microscopic slides and stained with DiffQuik staining kit. Differential cellular count was performed by standard morphological parameters in a single blinded manner. 200 leucocytes were counted from random fields. Data plotted as mean + SD. Data shown is a representative of four individual experiments using 4–6 mice in each group. Mann Whitney test was used to determine statistical significance. *p≤0.05 and **p≤0.01. Depletion of Tregs during the sensitization phase led to enhanced total cellular infiltration in BAL of DEREG mice on BALB/c genetic background also. Enhanced eosinophilia was observed in BAL from Treg depleted DEREG mice compared to their non-depleted counterparts. These results, confirm the potent regulatory influence of Tregs during the priming phase in both, allergy sensitive (BALB/c) and resistant (C57BL6) strain of mice.
(TIF)

## References

[1] Akdis M, Verhagen J, Taylor A, Karamloo F, Karagiannidis C, et al (2004) Immune responses in healthy and allergic individuals are characterized by a fine balance between allergen-specific T regulatory 1 and T helper 2 cells. J Exp Med 199: 1567–1575. 15173208 10.1084/jem.20032058 PMC2211782

[2] Hawrylowicz CM, O'Garra A (2005) Potential role of interleukin-10-secreting regulatory T cells in allergy and asthma. Nat Rev Immunol 5: 271–283. 15775993 10.1038/nri1589

[3] Curotto de Lafaille MA, Kutchukhidze N, Shen S, Ding Y, Yee H, et al (2008) Adaptive Foxp3+ regulatory T cell-dependent and -independent control of allergic inflammation. Immunity 29: 114–126. 18617425 10.1016/j.immuni.2008.05.010

[4] Hadeiba H, Locksley RM (2003) Lung CD25 CD4 regulatory T cells suppress type 2 immune responses but not bronchial hyperreactivity. J Immunol 170: 5502–5510. 12759427 10.4049/jimmunol.170.11.5502

[5] Baru AM, Hartl A, Lahl K, Krishnaswamy JK, Fehrenbach H, et al (2010) Selective depletion of Foxp3+ Treg during sensitization phase aggravates experimental allergic airway inflammation. Eur J Immunol 40: 2259–2266. 20544727 10.1002/eji.200939972

[6] Gri G, Piconese S, Frossi B, Manfroi V, Merluzzi S, et al (2008) CD4+CD25+ regulatory T cells suppress mast cell degranulation and allergic responses through OX40-OX40L interaction. Immunity 29: 771–781. 18993084 10.1016/j.immuni.2008.08.018 PMC2590499

[7] Meiler F, Klunker S, Zimmermann M, Akdis CA, Akdis M (2008) Distinct regulation of IgE, IgG4 and IgA by T regulatory cells and toll-like receptors. Allergy 63: 1455–1463. 18925882 10.1111/j.1398-9995.2008.01774.x

[8] Jaffar Z, Sivakuru T, Roberts K (2004) CD4+CD25+ T cells regulate airway eosinophilic inflammation by modulating the Th2 cell phenotype. J Immunol 172: 3842–3849. 15004191 10.4049/jimmunol.172.6.3842

[9] Joetham A, Takeda K, Taube C, Miyahara N, Matsubara S, et al (2007) Naturally occurring lung CD4(+)CD25(+) T cell regulation of airway allergic responses depends on IL-10 induction of TGF-beta. J Immunol 178: 1433–1442. 17237391 10.4049/jimmunol.178.3.1433

[10] Lewkowich IP, Herman NS, Schleifer KW, Dance MP, Chen BL, et al (2005) CD4+CD25+ T cells protect against experimentally induced asthma and alter pulmonary dendritic cell phenotype and function. J Exp Med 202: 1549–1561. 16314437 10.1084/jem.20051506 PMC2213331

[11] Suto A, Nakajima H, Kagami SI, Suzuki K, Saito Y, et al (2001) Role of CD4(+) CD25(+) regulatory T cells in T helper 2 cell-mediated allergic inflammation in the airways. Am J Respir Crit Care Med 164: 680–687. 11520737 10.1164/ajrccm.164.4.2010170

[12] Hall JA, Bouladoux N, Sun CM, Wohlfert EA, Blank RB, et al (2008) Commensal DNA limits regulatory T cell conversion and is a natural adjuvant of intestinal immune responses. Immunity 29: 637–649. 18835196 10.1016/j.immuni.2008.08.009 PMC2712925

[13] Lahl K, Loddenkemper C, Drouin C, Freyer J, Arnason J, et al (2007) Selective depletion of Foxp3+ regulatory T cells induces a scurfy-like disease. J Exp Med 204: 57–63. 17200412 10.1084/jem.20061852 PMC2118432

[14] Amu S, Gjertsson I, Tarkowski A, Brisslert M (2006) B-cell CD25 expression in murine primary and secondary lymphoid tissue. Scand J Immunol 64: 482–492. 17032240 10.1111/j.1365-3083.2006.01832.x

[15] Ardavin C, Shortman K (1992) Cell surface marker analysis of mouse thymic dendritic cells. Eur J Immunol 22: 859–862. 1347747 10.1002/eji.1830220334

[16] Couper KN, Lanthier PA, Perona-Wright G, Kummer LW, Chen W, et al (2009) Anti-CD25 antibody-mediated depletion of effector T cell populations enhances susceptibility of mice to acute but not chronic Toxoplasma gondii infection. J Immunol 182: 3985–3994. 19299696 10.4049/jimmunol.0803053 PMC3942880

[17] Setiady YY, Coccia JA, Park PU (2010) In vivo depletion of CD4+FOXP3+ Treg cells by the PC61 anti-CD25 monoclonal antibody is mediated by FcgammaRIII+ phagocytes. Eur J Immunol 40: 780–786. 20039297 10.1002/eji.200939613

[18] Hori S, Nomura T, Sakaguchi S (2003) Control of regulatory T cell development by the transcription factor Foxp3. Science 299: 1057–1061. 12522256 10.1126/science.1079490

[19] Chen GY, Chen C, Wang L, Chang X, Zheng P, et al (2008) Cutting edge: Broad expression of the FoxP3 locus in epithelial cells: a caution against early interpretation of fatal inflammatory diseases following in vivo depletion of FoxP3-expressing cells. J Immunol 180: 5163–5166. 18390696 10.4049/jimmunol.180.8.5163 PMC2527697

[20] Manrique SZ, Correa MA, Hoelzinger DB, Dominguez AL, Mirza N, et al (2011) Foxp3-positive macrophages display immunosuppressive properties and promote tumor growth. J Exp Med 208: 1485–1499. 21670203 10.1084/jem.20100730 PMC3135357

[21] Mayer CT, Kuhl AA, Loddenkemper C, Sparwasser T (2012) Lack of Foxp3+ macrophages in both untreated and B16 melanoma-bearing mice. Blood 119: 1314–1315. 22308282 10.1182/blood-2011-11-392266

[22] Put S, Avau A, Humblet-Baron S, Schurgers E, Liston A, et al (2012) Macrophages have no lineage history of Foxp3 expression. Blood 119: 1316–1318. 22308283 10.1182/blood-2011-11-391755

[23] Liston A, Farr AG, Chen Z, Benoist C, Mathis D, et al (2007) Lack of Foxp3 function and expression in the thymic epithelium. J Exp Med 204: 475–480. 17353370 10.1084/jem.20062465 PMC2137899

[24] Kim J, Lahl K, Hori S, Loddenkemper C, Chaudhry A, et al (2009) Cutting edge: depletion of Foxp3+ cells leads to induction of autoimmunity by specific ablation of regulatory T cells in genetically targeted mice. J Immunol 183: 7631–7634. 19923467 10.4049/jimmunol.0804308

[25] Lahl K, Sparwasser T (2011) In vivo depletion of FoxP3+ Tregs using the DEREG mouse model. Methods Mol Biol 707: 157–172. 21287334 10.1007/978-1-61737-979-6_10

[26] Kittel B, Ruehl-Fehlert C, Morawietz G, Klapwijk J, Elwell MR, et al (2004) Revised guides for organ sampling and trimming in rats and mice–Part 2. A joint publication of the RITA and NACAD groups. Exp Toxicol Pathol 55: 413–431. 15384248 10.1078/0940-2993-00349

[27] Cookson WO, Moffatt MF (2000) Genetics of asthma and allergic disease. Hum Mol Genet 9: 2359–2364. 11005790 10.1093/hmg/9.16.2359

[28] Suttner K, Depner M, Wetzke M, Klopp N, von Mutius E, et al (2010) Genetic variants harbored in the forkhead box protein 3 locus increase hay fever risk. J Allergy Clin Immunol 125: 1395–1399. 20398921 10.1016/j.jaci.2010.02.017

[29] Lluis A, Schedel M, Liu J, Illi S, Depner M, et al.. (2011) Asthma-associated polymorphisms in 17q21 influence cord blood ORMDL3 and GSDMA gene expression and IL-17 secretion. J Allergy Clin Immunol 127: 1587–1594 e1586. 10.1016/j.jaci.2011.03.015 21546069

[30] Kelada SN, Wilson MS, Tavarez U, Kubalanza K, Borate B, et al (2011) Strain-dependent genomic factors affect allergen-induced airway hyperresponsiveness in mice. Am J Respir Cell Mol Biol 45: 817–824. 21378263 10.1165/rcmb.2010-0315OC PMC3208613

[31] De Vooght V, Vanoirbeek JA, Luyts K, Haenen S, Nemery B, et al (2010) Choice of mouse strain influences the outcome in a mouse model of chemical-induced asthma. PLoS One 5: e12581. 20830207 10.1371/journal.pone.0012581 PMC2935354

[32] Navarro S, Cossalter G, Chiavaroli C, Kanda A, Fleury S, et al (2011) The oral administration of bacterial extracts prevents asthma via the recruitment of regulatory T cells to the airways. Mucosal Immunol 4: 53–65. 20811345 10.1038/mi.2010.51

[33] Wilson MS, Taylor MD, Balic A, Finney CA, Lamb JR, et al (2005) Suppression of allergic airway inflammation by helminth-induced regulatory T cells. J Exp Med 202: 1199–1212. 16275759 10.1084/jem.20042572 PMC2213237

[34] Dittrich AM, Erbacher A, Specht S, Diesner F, Krokowski M, et al (2008) Helminth infection with Litomosoides sigmodontis induces regulatory T cells and inhibits allergic sensitization, airway inflammation, and hyperreactivity in a murine asthma model. J Immunol 180: 1792–1799. 18209076 10.4049/jimmunol.180.3.1792

[35] Qiu H, Kuolee R, Harris G, Zhou H, Miller H, et al (2011) Acinetobacter baumannii infection inhibits airway eosinophilia and lung pathology in a mouse model of allergic asthma. PLoS One 6: e22004. 21789200 10.1371/journal.pone.0022004 PMC3138758

[36] Matsumoto K, Inoue H, Fukuyama S, Kan OK, Eguchi-Tsuda M, et al (2009) Frequency of Foxp3+CD4CD25+ T cells is associated with the phenotypes of allergic asthma. Respirology 14: 187–194. 19192224 10.1111/j.1440-1843.2008.01472.x

[37] Zuany-Amorim C, Haile S, Leduc D, Dumarey C, Huerre M, et al (1995) Interleukin-10 inhibits antigen-induced cellular recruitment into the airways of sensitized mice. J Clin Invest 95: 2644–2651. 7769104 10.1172/JCI117966 PMC295947

[38] Olszak T, An D, Zeissig S, Vera MP, Richter J, et al (2012) Microbial exposure during early life has persistent effects on natural killer T cell function. Science 336: 489–493. 22442383 10.1126/science.1219328 PMC3437652

[39] Arnold IC, Dehzad N, Reuter S, Martin H, Becher B, et al (2011) Helicobacter pylori infection prevents allergic asthma in mouse models through the induction of regulatory T cells. J Clin Invest 121: 3088–3093. 21737881 10.1172/JCI45041 PMC3148731

[40] D'Alessio FR, Tsushima K, Aggarwal NR, West EE, Willett MH, et al (2009) CD4+CD25+Foxp3+ Tregs resolve experimental lung injury in mice and are present in humans with acute lung injury. J Clin Invest 119: 2898–2913. 19770521 10.1172/JCI36498 PMC2752062

[41] Kearley J, Robinson DS, Lloyd CM (2008) CD4+CD25+ regulatory T cells reverse established allergic airway inflammation and prevent airway remodeling. J Allergy Clin Immunol 122: 617–624 e616. 10.1016/j.jaci.2008.05.048 PMC3389733 18672278

