# Supplementary figures and images for: Absence of Foxp3+ Regulatory T Cells during Allergen Provocation Does Not Exacerbate Murine Allergic Airway Inflammation
Source: PLoS One. 2012 Oct 10;7(10):e47102. doi: 10.1371/journal.pone.0047102 (PMC3468440; doi:10.1371/journal.pone.0047102)

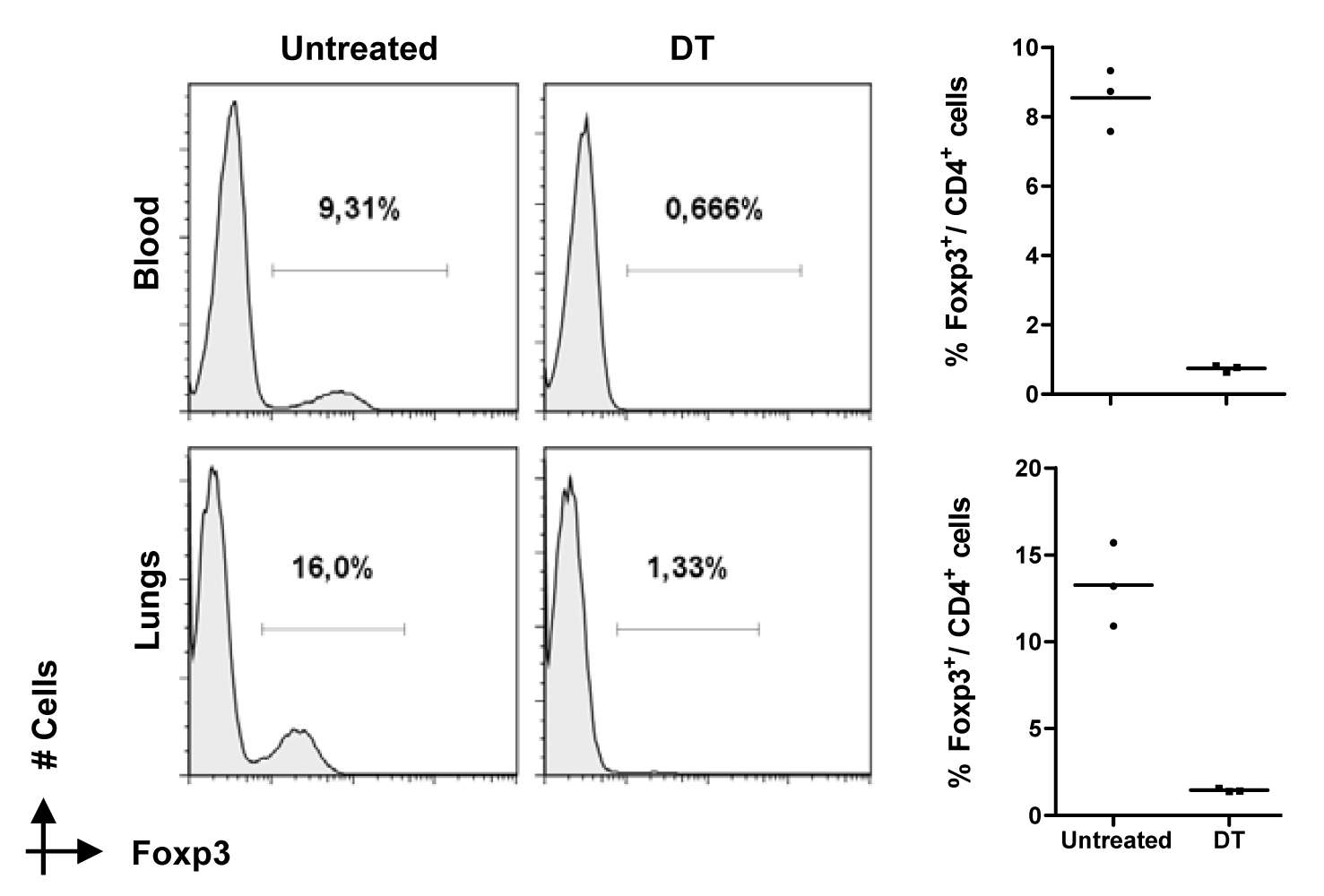

Supplement: Figure S1 — Depletion of Foxp3+ Tregs in lungs of DT treated mice. Since the sensitized and DT treated mice were to be challenged and as the lungs from these mice were used for mRNA extraction and histology, we used another set of mice to confirm depletion of Foxp3+ Tregs in lungs. DEREG-BALB/c mice received two i.p. injections of 0.5 µg DT on consecutive days and were sacrificed post 24 hrs of last treatment. Blood and lungs were collected and analyzed by FACS for Foxp3+ Tregs. Lungs were digested with collagenase D (Sigma-Aldrich, Munich, Germany) and DNAse I (Roche, Penzberg, Germany) and after RBC lysis blood and lung cells were stained for CD3, CD4 and intra-cellular Foxp3. Equivalent depletion of Foxp3+ Tregs was observed in lungs and blood as shown in the representative FACS histograms gated on live CD3+CD4+ T cells. Each dot in the graph represents data from individual mouse and horizontal bar indicate mean value. Data shown is a representative of two individual experiments with 3 mice per group. (TIF) [file pone.0047102.s001.tif]

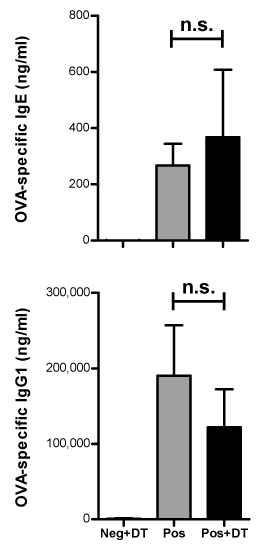

Supplement: Figure S2 — Sensitization remains unaffected by Foxp3+ Treg depletion during allergen provocation. To comprehend the influence of the absence of Tregs during the allergen challenge, it was essential to confirm an equivalent sensitization of mice in both the sensitized groups (Pos and Pos+DT). Individual sera samples were used to determine the titer of OVA-specific antibodies by ELISA. To measure OVA-specific IgE and IgG1, 10 µg/mL OVA (Grade V) (Sigma-Aldrich, Munich, Germany) was coated over night onto Maxisorp microtiter plates (Nunc, Roskilde, Denmark). After blocking with 1% BSA in PBS, samples and standards {IgG1: clone OVA-14 (Sigma-Aldrich, Munich, Germany) and IgE: clone-2C6 (AbD-Serotec, Oxford, UK)} were added and incubated over-night. Detection limits were 1.56 ng/mL and 3.125 ng/mL for IgE and IgG1, respectively. Bound antibodies were probed with biotinylated anti-mouse IgG1 (A85-1, BD Pharmingen, Heidelberg, Germany) or anti-mouse IgE (R35-92, BD Pharmingen, Heidelberg, Germany) for one hour at 37°C. Streptavidin conjugated alkaline phosphtase (Strep-AKP; BD Pharmingen, Heidelberg, Germany) was used to detect biotinylated antibodies. ELISA was developed with phosphatase substrate (Sigma-Aldrich, Munich, Germany) at room temperature. Absorbance was measured on the BioTek ELISA reader (Bad Friedrichshall Germany) at 450 nm with 570 nm filter as reference. Significant titers of OVA-specific IgE and IgG1 antibodies were detected in both the groups of mice sensitized to OVA in the presence of alum (Pos and Pos+DT). Antigen-specific IgE and IgG1 titers were at comparable levels irrespective of the Treg depletion status. As the sera were collected post allergen challenges, equivalent antigen-specific immunoglobulin levels validates that depletion of Tregs during allergen provocation did not affect the sensitization in the time frame of measurement i.e. from DT treatment to analysis. This further substantiates that the data presented in this study reflects the influence of [file pone.0047102.s002.tif]

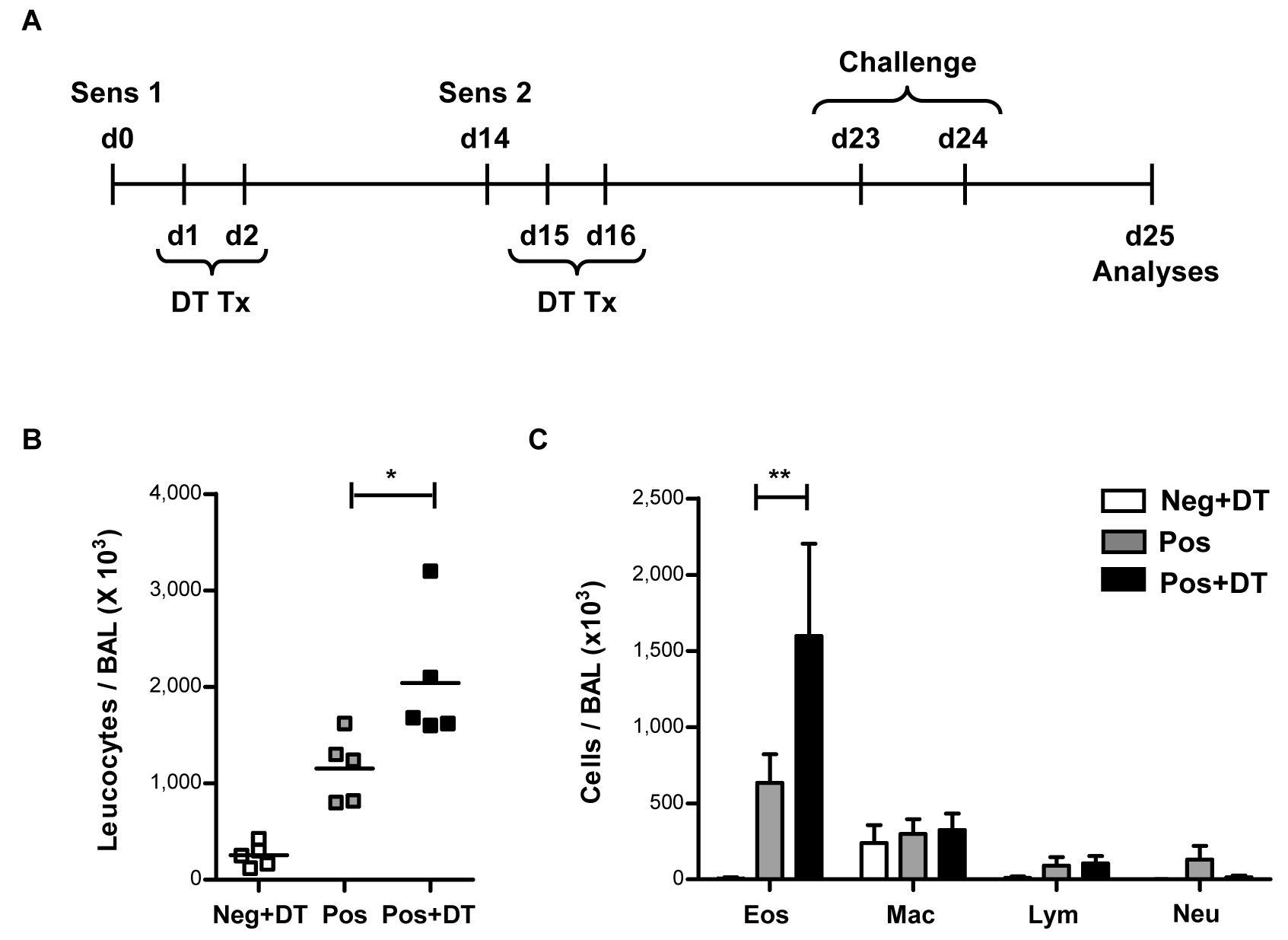

Supplement: Figure S3 — Depletion of Tregs during the sensitization phase exacerbates pathology in DEREG-BALB/c mice. We have recently reported that depletion of Tregs during the sensitization phase of allergic airway inflammation exacerbates lung pathology significantly using DEREG mice on C57BL/6 background. To assess the influence of strain specificity in absence of Tregs during the priming phase, we performed Treg ablation in DEREG mice on BALB/c genetic background during the sensitization phase. As BALB/c mice are comparatively allergy sensitive they were sensitized to OVA with alum by two i.p. injections one on day 0 and another on day 14. 0.5 µg DT was administered intra-peritoneally on two consecutive days after each sensitization to achieve Foxp3+ Tregs depletion during the sensitization phase. Mice were rested for one week after DT treatment, and then bled through retrobulbar venous plexus to confirm the rebound of Foxp3+ Tregs. Consequently, mice were challenged with OVA via intra-nasal route for two consecutive days and analyzed one day post last challenge. BAL was collected through the cannulated trachea with 0.8 ml PBS (3x). (A) Total cellular infiltration was estimated in the BAL by live cell counting with trypan blue exclusion dye. Each dot represents data from individual mice and horizontal line depicts mean value. (B) 5–10×104 BAL cells were spotted on microscopic slides and stained with DiffQuik staining kit. Differential cellular count was performed by standard morphological parameters in a single blinded manner. 200 leucocytes were counted from random fields. Data plotted as mean + SD. Data shown is a representative of four individual experiments using 4–6 mice in each group. Mann Whitney test was used to determine statistical significance. *p≤0.05 and **p≤0.01. Depletion of Tregs during the sensitization phase led to enhanced total cellular infiltration in BAL of DEREG mice on BALB/c genetic background also. Enhanced eosinophilia was observed in BAL from Treg depleted [file pone.0047102.s003.tif]
